# Urbanization Drives Habitat Suitability of the Invasive Cuban Knight Anole (*Anolis equestris*) in Florida, USA

**DOI:** 10.1002/ece3.72334

**Published:** 2025-10-14

**Authors:** Alexander S. Romer, Sergio A. Balaguera‐Reina, Eric Saurez, Edison D. Bonilla‐Liberato, W. James Whelpley, Frank J. Mazzotti, Melissa A. Miller

**Affiliations:** ^1^ Department of Wildlife Ecology and Conservation, Fort Lauderdale Research and Education Center University of Florida Davie Florida USA

**Keywords:** anthropogenic disturbance, citizen science, GBIF, habitat overlap, inaturalist, species distribution modeling, variable importance

## Abstract

This study evaluates climatic and anthropogenic drivers influencing habitat suitability of invasive Cuban knight anole ( *Anolis equestris* ) in Florida and assesses their potential impact on three species of threatened invertebrates due to habitat overlap. We developed species distribution models (SDMs) using eight algorithms to evaluate habitat suitability across the native and invasive range of *A. equestris* . We generated ten independent pseudo‐absence sets at a 1:1 ratio with presences and implemented a 10‐fold cross‐validation scheme. Predictor variables included effort, climatic, topographic, urbanization, and vegetation indices. We trained algorithms on 70% of the data, validated on 30%, constructed both algorithm‐specific and global ensembles. The best‐performing model was used to assess variable importance and predict habitat suitability across regions. Random Forest (RF) demonstrated the best overall performance (Florida: BI = 0.98, TSS = 0.91; Cuba: BI = 0.89, TSS = 0.74) and was used for subsequent analyses. When projected against an independent dataset with standardized effort, the model retained discriminatory power (TSS = 0.53; BI = 0.59), indicating generalizability. Mean diurnal range was the most influential predictor overall, while urbanization (e.g., settlement model grid) was more important in Florida. NDVI and precipitation of the driest month had greater influence in Cuba. Predicted habitat suitability at occurrence locations of the endangered Florida tiger beetle (*Cicindelidia floridana*, *x̄* = 0.86), Florida tree snail ( *Liguus fasciatus* , *x̄* = 0.58), and endangered Schaus' swallowtail butterfly ( *Papilio aristodemus* , *x̄* = 0.53), suggest potential overlap. These findings emphasize the role of urbanized habitats in facilitating invasion and provide a data‐driven framework for conservation management and mitigation.

## Graphical abstract

This study evaluates the climatic and anthropogenic factors driving the habitat suitability of the invasive Cuban knight anole ( *Anolis equestris* ) in Florida and its potential impact on three threatened invertebrate species through habitat overlap. Using species distribution models (SDMs), we found that urbanization strongly influences the anole's invasive distribution, with high suitability in South Florida and the Keys. Our results highlight spatial overlap between *A. equestris* and imperiled invertebrates, suggesting potential predation risk, and emphasize the role of urbanization in facilitating biological invasions.

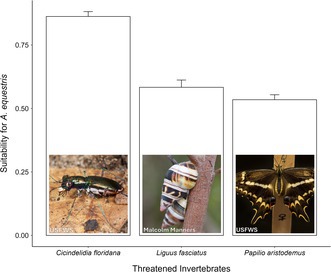

## RESUMEN

Determinar qué factores climáticos y antropogénicos influyen en la idoneidad de hábitat del chipojo ( *Anolis equestris* ) como especie invasora en Florida y analizar su posible impacto sobre tres especies de invertebrados amenazados. Se desarrollaron modelos de distribución de especies (SDM) utilizando ocho algoritmos para evaluar la idoneidad del hábitat en la distribución actual de *A. equestris*. Generamos diez conjuntos independientes de pseudo‐ausencias en una proporción 1:1 con las presencias e implementamos un esquema de validación cruzada de 10 pliegues. Las variables predictoras incluyeron esfuerzo, clima, topografía, urbanización e índices de vegetación. Entrenamos los algoritmos con el 70% de los datos, los validamos con el 30%, y construimos tanto ensamblajes específicos de algoritmos como ensamblajes globales. Se utilizó el mejor modelo para evaluar la importancia de las variables y predecir la idoneidad del hábitat en las diferentes regiones. El algoritmo Random Forest (RF) demostró el mejor desempeño general (Florida: BI = 0.98, TSS = 0.91; Cuba: BI = 0.89, TSS = 0.74) y fue utilizado en los análisis posteriores. Al proyectarse sobre un conjunto de datos independiente con esfuerzo estandarizado, el modelo mantuvo su capacidad discriminativa (TSS = 0.53; BI = 0.59), lo que indica su capacidad de generalización. El rango diurno promedio fue el predictor más influyente en general, mientras que la urbanización (por ejemplo, la cuadrícula del modelo de asentamientos) tuvo mayor importancia en Florida. El NDVI y la precipitación del mes más seco mostraron mayor influencia en Cuba. La idoneidad del hábitat predicha en las localidades de ocurrencia del escarabajo tigre de Florida en peligro de extinción (*Cicindelidia floridana*, *x̄* = 0.86), el caracol arbóreo de Florida ( *Liguus fasciatus* , *x̄* = 0.58), y la cometa caribeña en peligro de extinción ( *Papilio aristodemus* , *x̄* = 0.53), sugiere un posible solapamiento. Estos hallazgos denotan la influencia positiva de los hábitats urbanizados en los procesos de colonización de las especies y proporcionan un marco basado en datos para la gestión de la conservación y estrategias de mitigación.

## 1 Introduction

Invasive species are a primary driver of global biodiversity decline, altering ecosystem structure and function through mechanisms including predation, competition, and habitat modification (Simberloff et al. 2013). However, the impacts of invasive species can be mitigated through predictive modeling (Chai et al. 2016). By forecasting potential invasion hotspots, predictive models facilitate Early Detection and Rapid Response (EDRR), which reduces ecological damage and economic costs compared to managing established invasions (García‐de‐Lomas et al. 2022; Westbrooks et al. 2022). These models also inform targeted management strategies, such as habitat modification and monitoring programs, that help control invasive species before they become widespread (Lyons et al. 2020; Meriggi et al. 2022). Species distribution models (SDMs) encompass a range of quantitative techniques that use environmental data to predict species' probability of occurrence (e.g., habitat suitability) or abundance (Elith and Leathwick 2009). SDMs support effective management of non‐native species by providing insight into the relationship between environmental factors and the occurrence of introduced species, enabling the delineation of potential invasion extent (Chapman et al. 2019; Lozano et al. 2024). However, the utility of these models depends on a considered approach and an awareness of their limitations.

The application of SDMs to invasive species necessarily invokes discussion of how native and invasive occurrence data should be integrated. How these data are handled in the implementation of SDM algorithms can significantly affect the accuracy and reliability of their predictions. Lake et al. (2020) demonstrated that using occurrence datasets solely from the non‐native range of a species can lead to spatial biases and divergent model outputs. Furthermore, several studies have found that incorporating both native and introduced occurrence data results in more accurate model projections (Chapman et al. 2019; Mainali et al. 2015). Despite this benefit, collecting occurrence data throughout a species' native and invasive range may be logistically difficult or prohibitively expensive. Thus, the increasing availability of data through community science platforms like The Global Biodiversity Information Facility (GBIF) can increase the utility of SDMs for invasive species (Di Cecco et al. 2021; Feldman et al. 2021).

The GBIF provides streamlined access to biodiversity records, including community science observations, museum specimens, and institutional datasets (Telenius 2011). By accessing these resources, investigators can integrate a large volume of occurrence data into SDMs with low resource expenditure. However, the use of such data must be carefully managed, as quality assurance/quality control and validation are essential to ensure reliable predictions and inference (Dickinson et al. 2010). Previous studies have successfully incorporated community science data into SDMs by creating composite datasets from internally generated data (Robinson et al. 2020; Roy‐Dufresne et al. 2019). Another approach involves fitting SDMs with community science data and validating resulting models using independent, internally collected datasets (Matutini et al. 2021). The latter approach offers significant advantages: using community science data expands the spatial coverage and sampling depth of the models, while validating predictions with high‐quality, independent data ensures robust model support. As such, this technique offers a powerful framework for modeling the spread of invasive species, particularly in groups with limited prior application of species distribution models such as the anole lizards (family Dactyloidae).

The family Dactyloidae contains approximately 400 species of neo‐tropical lizards (Nicholson et al. 2012). Several of its members, including the brown anole ( *Anolis sagrei* ) and the green anole ( *Anolis carolinensis* ), have become established outside their natural range (Fisher et al. 2020; Mayer and Lazell 2021; Norval et al. 2012, 2016). Their success as invaders is attributed to several factors, including behavioral plasticity that allows them to adapt to a wide variety of environments (Jackson and Bleicher 2024), high reproductive rates that enable rapid population growth (Fetters and McGlothlin 2017), and human‐mediated dispersal, which facilitates their movement across geographical barriers (Kolbe et al. 2017). Additionally, urbanization imposes distinct selective pressures on anoles, with consequences for their morphology and behavior (Lailvaux 2020; Thawley et al. 2019). For example, invasive *A. sagrei* exhibit behavioral adaptations to urban environments, including increased use of artificial substrates and more frequent dewlap displays, which facilitate intraspecific interactions in open habitats (Stroud et al. 2019). In the Cuban knight anole ( *Anolis equestris* ), invasion success appears to depend on the environmental context, particularly between natural and urban settings.

Endemic to Cuba, *A. equestris* was first introduced to South Florida in the 1950s due to intentional release (Camposano et al. 2008; Losos 2011). As of 2019, *A. equestris* had been recorded in 14 Florida counties, expanding its range along the Atlantic coastal ridge, as well as into Southwest Florida and the Florida Keys (Camposano and Krysko 2019). These records demonstrate a northernmost extent for the species in St. John's County (Camposano and Krysko 2019). In Cuba, *A. equestris* occupies a broad environmental niche, including arboreal habitats in moist forests, plantations, and urban green spaces (Losos 2011). However, in Florida, *A. equestris* predominantly inhabits urban and suburban areas, with limited presence in low‐disturbance habitats like Everglades National Park (EDDMapS 2025). This distribution suggests a potential niche shift towards urban environments in the invasive range. Similar shifts towards anthropogenic habitat have been observed in invasive populations of plants (González‐Moreno et al. 2015), birds (Cardador and Blackburn 2020), and insects (Hill et al. 2017; Menke et al. 2007). However, a meta‐analysis found an overall negative association between habitat modification and reptile populations, including in the family Dactyloidae (Doherty et al. 2020). As previously described, invasive populations of *Anolis* spp. display unique behavioral and morphological responses to anthropogenic environments. For example, *A. sagrei* shows increased growth and reproductive output around nocturnal artificial lighting (Thawley and Kolbe 2020). Thus, utilization of anthropogenic habitats may influence reptiles' capacity to successfully establish invasive populations. Understanding the drivers of *A. equestris* ' distribution in their invasive and native ranges may elucidate factors that contribute broadly to invasiveness in reptiles.

In plant ecology, exotic species are often conceptualized along a continuum of introduction–naturalization–invasion (Richardson et al. 2000; Richardson and Pyšek 2012). Introduction occurs when an exotic species is transported across a dispersal barrier by human activities, while naturalization refers to a species' ability to reproduce consistently in the new environment (Richardson et al. 2000). Invasion is defined by the distance reproductive populations disperse from the introduction site (Richardson et al. 2000). However, a mechanistic definition of biological invasion encompasses high rates of proliferation, competitive advantage over native species, and rapid spread into novel environments (Valéry et al. 2008). We adhere to the latter definition of “invasive”. Under this paradigm, exotic species are categorized as either naturalized, meaning they have low ecological impact, or invasive, meaning they have significant ecological impact. *Anolis equestris* is not currently documented to exhibit traits associated with invasive species, such as impacting native biodiversity. *Anolis equestris* has not received any special designation as an exotic species by Florida environmental agencies (SFWMD and USACE 2024). Thus, it is typically considered a naturalized species with low priority for management. However, synanthropy does not preclude invasiveness among exotic vertebrates (Banks and Smith 2015), particularly when contact with threatened native species occurs (Wells et al. 2014).

*Anolis equestris* exhibits an omnivorous and opportunistic diet in both its native and introduced ranges. In Cuba, its diet includes a diverse array of invertebrates, fruits, seeds, and occasional vertebrate prey such as smaller anoles and tree frogs (Rodriguez Schettino 1999). In Florida, its diet is similarly diverse, with the insectivorous portion of its diet primarily consisting of Coleopterans and Lepidopterans (Dalrymple 1980). Invasive populations also consume gastropods such as the Florida tree snail ( *Liguus fasciatus* ) (Giery et al. 2017). While prior studies have not directly examined the impact of *A. equestris* on threatened invertebrates in Florida, this dietary plasticity raises concerns. For example, the federally endangered Schaus swallowtail butterfly ( *Papilio aristodemus* ), as well as *L. fasciatus* , formerly a state threatened species (FWC 2016; delisted in 2016), are potential prey. Urbanization exacerbates these risks by fragmenting habitats and reducing spatial buffers at the wildland–urban interface, increasing interactions between invasive and native species (Bar‐Massada et al. 2014). For instance, the Miami tiger beetle (*Cicindelidia floridana*), a federally endangered species restricted to urban pine rocklands in Miami‐Dade County, may face predation pressure from *A. equestris* (Knisley and Brzoska 2018). As such, *A. equestris* may pose a previously unrecognized threat to native biodiversity as urban development continues across South Florida.

In this study, we employed species distribution modeling (SDM) to assess the occurrence of *A. equestris* across both its native range (Cuba) and its introduced range (Florida). We treat SDM outputs as an approximation of the realized environmental niche (e.g., the abiotic conditions the species occupies in its accessible range) in the study region (Soberón and Peterson 2005). By modeling under current climatic and land‐use conditions, our approach identifies the presently invasible area, producing a near‐term risk map of habitats suitable now or in the immediate future (Hui 2023). Specifically, our objectives were to: (1) predict habitat suitability for *A. equestris* in both regions; (2) evaluate the relative importance of environmental factors influencing its distribution in each region; (3) validate model predictions using independent data from long‐term monitoring of invasive reptiles in South Florida; and (4) determine the potential for *A. equestris* to spatially overlap with three protected invertebrates: the Florida tree snail ( *Liguus fasciatus* ), the Miami blue butterfly ( *Papilio aristodemus ponceanus* ), and the Miami tiger beetle (*Cicindelidia floridana*). All are listed under state or federal conservation statutes, occur within *A. equestris'* current invasion extent, and represent groups consumed by *A. equestris* in Florida (Dalrymple 1980; Giery et al. 2017). We predict that potential habitat suitability for *A. equestris* can be accurately identified with high accuracy by integrating environmental data from both ranges into SDMs. We hypothesize that in its native range, the distribution of *A. equestris* will be primarily driven by climatic and land cover factors, whereas in its invasive range, anthropogenic factors (e.g., urbanization) will play a greater role. We predict that independent survey data of *A. equestris* in South Florida will validate model projections. Finally, we predict that the habitat of threatened native invertebrates will be suitable for *A. equestris* , indicating the potential for overlap and interaction. By clarifying the drivers of *A. equestris* ' distribution across its native and invasive ranges, this study aims to elucidate factors that promote invasiveness in reptiles.

## 2 Methods

### 2.1 Study Area and Data Collection

We modeled the distribution of *A. equestris* across its native range in Cuba and invasive range in Florida, USA. Shapefiles representing the geographic boundaries of Florida and Cuba were used to define the study's extent. The Florida state boundary shapefile was obtained from the Florida Department of Transportation (published March 13, 2019; https://hub.arcgis.com/datasets/519e0a0ed5984bedba53695e1f56c1ee), while the Cuba Subnational Administrative Boundaries shapefile was sourced from the United Nations Office for the Coordination of Humanitarian Affairs (published June 21, 2019; https://data.humdata.org/dataset/cod‐ab‐cub). These shapefiles were processed in R (version 4.4.1) using the *terra* and *sf* packages (Hijmans 2024; Pebesma 2018).

We accessed occurrence records for *A. equestris* collected between 01/01/1900 and 12/04/2024 from the Global Biodiversity Information Facility (GBIF) using the *rgbif* package (Chamberlain and Boettiger 2017). Spatial precision differed markedly between regions. In Florida (*n* = 1941), 82% of records had a coordinate uncertainty (CU) ≤ 1000 m, with a median CU of 31 m. In contrast, among Cuban records (*n* = 172), only 52% had a CU ≤ 1000 m, and the median CU was 900 m. Characteristics of the data contributing to this disparity include collection date and record provenance. In Florida, 96% of records were collected after 2000, approximately corresponding to the widespread adoption of handheld GPS devices, compared to only 36% of Cuban records. Additionally, 65% of Cuban records were associated with preserved museum specimens, which are often retrospectively georeferenced to locality centroids and thus have higher positional uncertainty (Murphey et al. 2004). By contrast, only 8% of Florida records were museum specimens. Applying a consistent CU cutoff of 1000 m would have excluded over 60% of native‐range records, greatly reducing ecological representativeness in Cuba. To balance spatial precision with data retention, we applied region‐specific thresholds: all Florida records with CU > 1 km were excluded (retained *n* = 1941, *x̄* = 67 ± 134 m, range: 1–1000 m), as were Cuban records with CU > 15 km (retained *n* = 166, *x̄* = 2735 ± 3698 m, range: 4–14,977 m). All records were screened using the *CoordinateCleaner* package, which applied tests for erroneous coordinates, such as the removal of records with non‐terrestrial locations (Zizka et al. 2019). We subsampled occurrence data so that only one presence record was retained per raster cell (30 arc sec, ~1 km^2^, WGS 84). After subsampling, the final dataset comprised a total of 1012 presence records: 940 from Florida and 72 from Cuba.

### 2.2 Environmental Variables

We selected environmental predictors to capture the climatic, topographic, and anthropogenic factors influencing *A. equestris* habitat suitability. Nineteen Bioclimatic variables, representing long‐term means of temperature and precipitation at a spatial resolution of 30 arc sec, were sourced from the WorldClim database version 2.1 (Fick and Hijmans 2017). Elevation data were obtained from the Global Multi‐resolution Terrain Elevation Data 2010 (GMTED2010) dataset (Danielson and Gesch 2011). Urbanization metrics were sourced from the Global Human Settlement Layers dataset, including built‐up height, built‐up surface, built‐up volume, population density, and settlement model grids (Melchiorri 2022). These variables quantified anthropogenic modifications and human population within the study area. Additionally, surface temperature, Enhanced Vegetation Index (EVI), and Normalized Difference Vegetation Index (NDVI) layers were sourced from Moderate Resolution Imaging Spectroradiometer (MODIS) data (MODIS Science Team 2021a, 2021b). All environmental raster layers were defined with the WGS 84 coordinate system, resampled to 30 arc sec, centered, and scaled.

Additionally, to account for spatial sampling bias associated with community science data, we derived a sampling‐effort surface from iNaturalist records. We downloaded all occurrence records for organisms within the Phylum Chordata in Cuba and Florida from iNaturalist on 11/26/2024. Points were rasterized using the same spatial framework (datum, pixel size, extent) as the other environmental layers. We defined the value of each raster cell using a custom function that assigned each cell the natural logarithm of the number of observations it contained. We assigned a value of 0.33 to cells with one observation and a value of 0 to cells with no observations to avoid compressing low‐effort cells to the same value as unsampled cells. No additional centering or scaling was applied to this raster, as the log transformation suitably constrained its range. This layer was included as an environmental covariate in all model fits. During projection on non‐GBIF data, it was set to the mean of all non‐zero effort values (1.16) to allow for model predictions of climatic, topographic, and anthropogenic effects on suitability while holding observer bias constant.

To address multicollinearity among predictors, we calculated pairwise Pearson correlation coefficients and excluded variables with correlation coefficients exceeding |0.7|. We retained 11 layers after this procedure: Land Fraction Pixel, NDVI, Elevation, Precipitation of Driest Month, Precipitation of Wettest Quarter, Surface Temperature, Annual Precipitation, Settlement Model Grid, Population Grid, Mean Diurnal Range, and Inaturalist Effort. Further details on these layers, including native resolutions and descriptions, are provided in Table S1.

### 2.3 Pseudo‐Absence Generation

To address the limitations of presence‐only data in species distribution modeling, we generated sets of pseudo‐absence (PA) points at a 1:1 ratio with presences on a per‐country basis [e.g., *n* = 72 for Cuba] (Whitford et al. 2024). We generated ten independent PA sets, each produced under a unique random seed, to reduce the stochasticity associated with absence selection in model selection and fitting (Barbet‐Massin et al. 2012). The method used to generate these points differed slightly between *A. equestris* ' native and invasive range to accommodate variation in measurement precision.

In the invasive range (Florida), all occurrence records had positional uncertainties no greater than a single raster cell (1 km). Raster cells containing a presence record were excluded from PA point generation. However, neighboring raster cells were viable locations for pseudo‐absence points. The ten PA sets comprised a total of 9400 PA points (Figure S1). In the native range (Cuba), a subset of occurrence records (*n* = 29) had positional uncertainties greater than 1 km, spanning multiple raster cells. To account for this, we generated a buffer around the reported locations, encompassing the extent of the positional uncertainty radius. Raster cells intersecting or contained within these buffers were excluded from PA point generation (Figure S2). This conservative technique minimizes the risk of generating PA points in areas associated with occurrence records. Such an approach is expected to increase the performance of SDMs when the measurement error of positional data is higher than raster cell resolution (Naimi et al. 2014; VanDerWal et al. 2009). Ten PA sets comprising a total of 720 PA points (Figure S2) were generated using this technique.

### 2.4 Model Training and Cross‐Validation

Presence records from GBIF were merged with each of the ten PA sets, giving ten balanced data sets that differed only in the background points against which the fixed set of presences was contrasted (Barbet‐Massin et al. 2012). The combined data were first stratified by country and class and then split into training (70%) and validation (30%) partitions. Random seeds were set for data partitioning to ensure reproducibility. The training dataset was used for model fitting procedures as described below. The validation dataset was used as a semi‐independent measure of model performance in that it was withheld from model training, but it was derived from the same source data.

We implemented Species distribution modeling using the *biomod2* package (Thuiller et al. 2024). We selected eight modeling algorithms for evaluation: Artificial Neural Networks (ANN), Classification Tree Analysis (CTA), Flexible Discriminant Analysis (FDA), Multivariate Adaptive Regression Splines (MARS), Random Forest (RF), Gradient Boosting Machines (GBM), Extreme Gradient Boosting (XGBoost), and Maximum Entropy (MAXENT). Hyperparameters for each algorithm were selected using the “bigboss” configuration provided within *biomod2*. These parameters are pre‐optimized for robust performance across diverse modeling scenarios. Class weights were unnecessary because prevalence was held at 0.5 and observer bias was captured explicitly by the iNaturalist effort layer.

Because individual algorithms can yield divergent predictions, especially when sample sizes are small and background selection varies (Liu et al. 2019), we adopted an ensemble approach to improve stability and accuracy. For each algorithm, we first built an algorithm‐level ensemble by averaging predictions from all cross‐validation folds across the ten PA sets by taking the unweighted arithmetic mean of predicted probabilities across runs (*EMmean*). This reduces variability introduced by different background samples while keeping the modeling assumptions of each algorithm intact. We also generated three global ensembles using *EMmean*, *EMca* (committee averaging), and *EMwmean* (TSS‐weighted mean) to pool predictions across all algorithms, cross‐validation folds, and PA sets. Global ensembles dampen individual‐model biases and can enhance spatial transferability (Marmion et al. 2009).

We evaluated the performance of all ensemble models, per‐algorithm and global, using the validation partition of each PA set. The True Skill Statistic (TSS) was used to evaluate model discrimination, while the Boyce index (BI) was used to assess how effectively models distinguished observed presence locations from withheld PA (i.e., background) points based on predicted suitability. The BI measures the correlation between predicted suitability and observed presence frequencies, offering insight into the ecological plausibility of the model predictions (Hirzel et al. 2006). Validation metrics were calculated separately for Florida and Cuba to assess regional variation in performance. We then selected the ensemble with the highest joint mean of TSS and BI as the final model for subsequent variable importance analyses and generation of habitat suitability maps for *A. equestris* in its native and invasive ranges.

### 2.5 Variable Importance

We assessed variable importance using the permutation‐based approach provided in *biomod2* (Thuiller et al. 2024). This method calculates the Pearson's correlation between predictions made with the original data and predictions made with a shuffled variable of interest, then an importance is derived as $1-\mathrm{cor}\left(\text{reference},\text{shuffled}\right)$. A higher score indicates that the shuffled variable had a greater impact on model predictions, meaning it is more influential in the model. A score of 0 indicates that the variable had no impact when shuffled, suggesting no influence on model predictions.

We extracted importance scores from the full set of 100 constituent models (ten pseudo‐absence sets × ten cross‐validation folds) comprising our final ensemble model. To identify regional differences in predictor importance, variable importance was calculated separately for validation data corresponding to the native (Cuba) and invasive (Florida) ranges, using only the first PA validation set. For each variable in each constituent model, we shuffled predictor values nine times and computed importance scores by measuring reductions in predictive accuracy. The resulting raw importance values were then standardized to sum to one within each model run and region combination, ensuring that scaled importance scores were comparable across predictors and regions.

To test whether the relative influence of predictors differed between Cuba and Florida, we fitted a beta‐regression mixed model with *glmmTMB* (Brooks et al. 2017). The model formula specified region (Cuba vs. Florida), predictor, and their interaction as fixed effects, while algorithm‐run was included as a random intercept to account for non‐independence among replicates. Diagnostic checks indicated heteroscedasticity, so we specified an identical dispersion formula (~ region × predictor) to allow residual variance to vary across region–predictor combinations. Estimated marginal means, representing the average scaled importance of each variable, were calculated using the *emmeans* package (Lenth 2024). Pairwise contrasts of these estimated marginal means were calculated to identify significant differences in variable importance between Cuba and Florida.

### 2.6 Long‐Term Monitoring Program

A long‐term (2008–2023) monitoring effort for invasive ectotherms in South Florida was conducted using surveys performed monthly along designated routes to detect and monitor populations of invasive ectotherms. This monitoring program utilizes vehicular and visual encounter survey (VES) techniques to collect data.

During surveys, biologists traverse the route at ~25–50 kph using motor vehicles, recording any observations of invasive or native reptiles. At the beginning and end of each survey route, VESs are performed on foot. These consist of a five‐minute search of the area within a 100‐m radius of a pre‐designated location (31,416 m^2^) by a pair of trained observers. Survey routes can have between zero and three additional VES locations. If VESs are conducted at night, Fenix headlamps (Fenix Lighting, Littleton, CO, USA) are used to aid in the detection of reptiles and amphibians. Coordinates of records were collected using GARMIN GPS 64× handheld devices (GARMIN International, Olathe, KS, USA; accuracy ±3). This methodology was adapted from protocols that have been demonstrated to be effective in surveying for reptiles and amphibians in South Florida (Bernardino and Dalrymple 1992; Waddle et al. 2010).

We utilized data from this monitoring program database to conduct independent validation of the model with the highest performance on the internal validation dataset. This quantified the model's ability to predict presences and absences in an independent dataset, providing a strong measure of generalizability. Records with missing or invalid spatial coordinates, duplicate entries, or uncertain identifications were removed. We selected VES locations which had been surveyed at least 25 (*n* = 106) times to minimize the chance of recording false negatives. For each of these locations, a site was recorded as positive for the presence of *A. equestris* if the species had ever been observed there (*n* = 8) and negative otherwise (*n* = 98). Additionally, if *A. equestris* was observed while traversing the survey route, it was included as a positive occurrence point (*n* = 98). To minimize spatial autocorrelation, we aggregated the combined checkpoint and route records to the 1‐km grid used for model projections, retaining at most one point per cell and giving precedence to presences when both classes occurred in the same cell. This subsampling yielded 104 evaluation points (23 presences and 81 absences) that were used for the independent assessment of model accuracy. The True Skill Statistic (TSS) and BI were utilized to assess predictive accuracy. A map of all observations in this dataset can be found in Figure S3.

### 2.7 Threatened Invertebrates

Using the model selected by our a‐priori criteria, we evaluated habitat overlap between *A. equestris* and three threatened invertebrate species, recognizing that direct interactions remain uncertain due to potential differences in microhabitat. These species were *L. fasciatus* , *P. aristodemus* , and *C. floridana* . Both *P. aristodemus* and *C. floridana* are currently federally endangered. Occurrence data for *L. fasciatus* (*n* = 223) were generated by surveys conducted by the Florida Fish and Wildlife Conservation Commission (FWC) from 2015 to 2020. Occurrence data for *P. aristodemus* (*n* = 542) were generated by surveys conducted by the Florida Department of Environmental Protection (FDEP) from 2021 to 2022. We derived occurrence points for *C. floridana* by calculating the centroid for each of 6 species management units within the Richmond Heights pine‐rocklands (mean unit area = 0.15 km^2^; Knisley and Brzoska (2018)).

To ensure consistency with the *A. equestris* occurrence data, we subsampled points for each invertebrate species such that only one occurrence point per raster cell was retained. This procedure reduced the dataset to 5 points for *C. floridana* , 5 points for *L. fasciatus* , and 37 points for *P. aristodemus* . Habitat suitability for *A. equestris* was predicted at each raster cell containing invertebrate occurrence points using the final ensemble model of *A. equestris* occurrence (see Model Training and Cross‐Validation) of *A. equestris* occurrence. Suitability scores at these cells were extracted and averaged for each species. Standard errors were calculated to quantify the variability in suitability scores across occurrence points within each species.

## 3 Results

### 3.1 Model Performance

**FIGURE 1:**
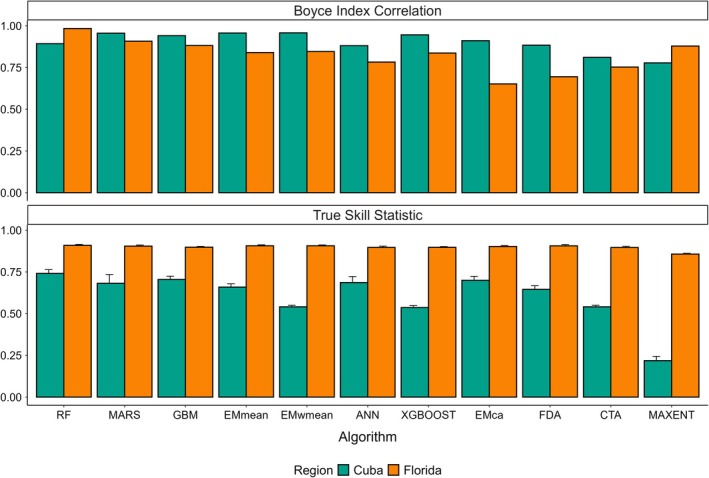
Boyce Index (BI) and True Skill Statistic (TSS) for species distribution models of *Anolis equestris* . Bars represent mean values of BI and TSS for each model. Error bars (± standard error) are shown only for TSS, which was calculated across ten pseudo‐absence sets; BI was computed on a fixed set of presence locations and does not include error estimates. Algorithm abbreviations: ANN, artificial neural network; CTA, Classification Tree Analysis; EMca, committee averaging; EMmean, unweighted mean; EMwmean, TSS‐weighted mean; FDA, Flexible Discriminant Analysis; GBM, gradient boosted machine; MARS, multivariate adaptive regression splines; Maxent, maximum entropy; RF, random forest; XGBOOST, Extreme Gradient Boosting.

Among the ensemble models we evaluated, Random Forest (RF), Multivariate Adaptive Regression Splines (MARS), Gradient Boosting Machines (GBM), unweighted mean (EMmean), and TSS‐Weighted Mean (EMwmean) demonstrated the highest performance across Florida and Cuba on the validation set (Figure 1). RF demonstrated the best overall performance, achieving a BI of 0.98 and a TSS of 0.91 in Florida, and a BI of 0.89 and a TSS of 0.74 in Cuba. MARS performed well in Florida with a BI of 0.91 and a TSS of 0.90, but with reduced discrimination performance in Cuba, with a BI of 0.96 and a TSS of 0.68. GBM also performed strongly in Florida with a BI of 0.88 and a TSS of 0.90. However, as with MARS, this model struggled to generalize to Cuba, with a BI of 0.94 and a TSS of 0.70. Of the global ensembles, EMmean produced a BI of 0.84 and a TSS of 0.91 in Florida and a BI of 0.96 and a TSS of 0.66 in Cuba. The TSS‐weighted ensemble (EMwmean) showed similar performance in Florida (BI = 0.85, TSS = 0.91) but demonstrated lower discriminatory power in Cuba (BI = 0.96, TSS = 0.54). A complete list of validation metrics for all ensemble models and both regions is provided in Table S2.

Following the a priori selection criteria described in the methods, we proceeded with RF as our final model. To assess how standardizing the sampling effort layer influenced model performance, we projected the final model onto the GBIF‐derived test dataset after replacing the original effort values with their mean non‐zero value (1.16). Standardizing sampling effort caused only minor decreases in predictive performance: TSS shifted from 0.74 to 0.57 in Cuba and from 0.91 to 0.89 in Florida, while the BI changed from 0.89 to 0.96 in Cuba and from 0.98 to 0.85 in Florida. Subsequently, we assessed the generalizability of the standardized effort projection using data from the long‐term invasive reptile monitoring program, which was unaffected by citizen‐science sampling biases. On this fully independent validation set, the model achieved moderate predictive performance (BI = 0.59, TSS = 0.53), with an overall accuracy of 0.86 (95% CI: 0.78–0.92), sensitivity of 0.94, specificity of 0.59, Kappa of 0.56, and balanced accuracy of 0.76. Together, these results indicate that the final model retains predictive utility when generalized to both semi‐independent (GBIF‐derived) and fully independent datasets. Importantly, the limited reduction in predictive performance upon standardizing sampling effort demonstrates that the model robustly captures the influence of environmental variables on habitat suitability, rather than primarily relying on sampling effort bias.

### 3.2 Native Habitat Suitability

**FIGURE 2:**
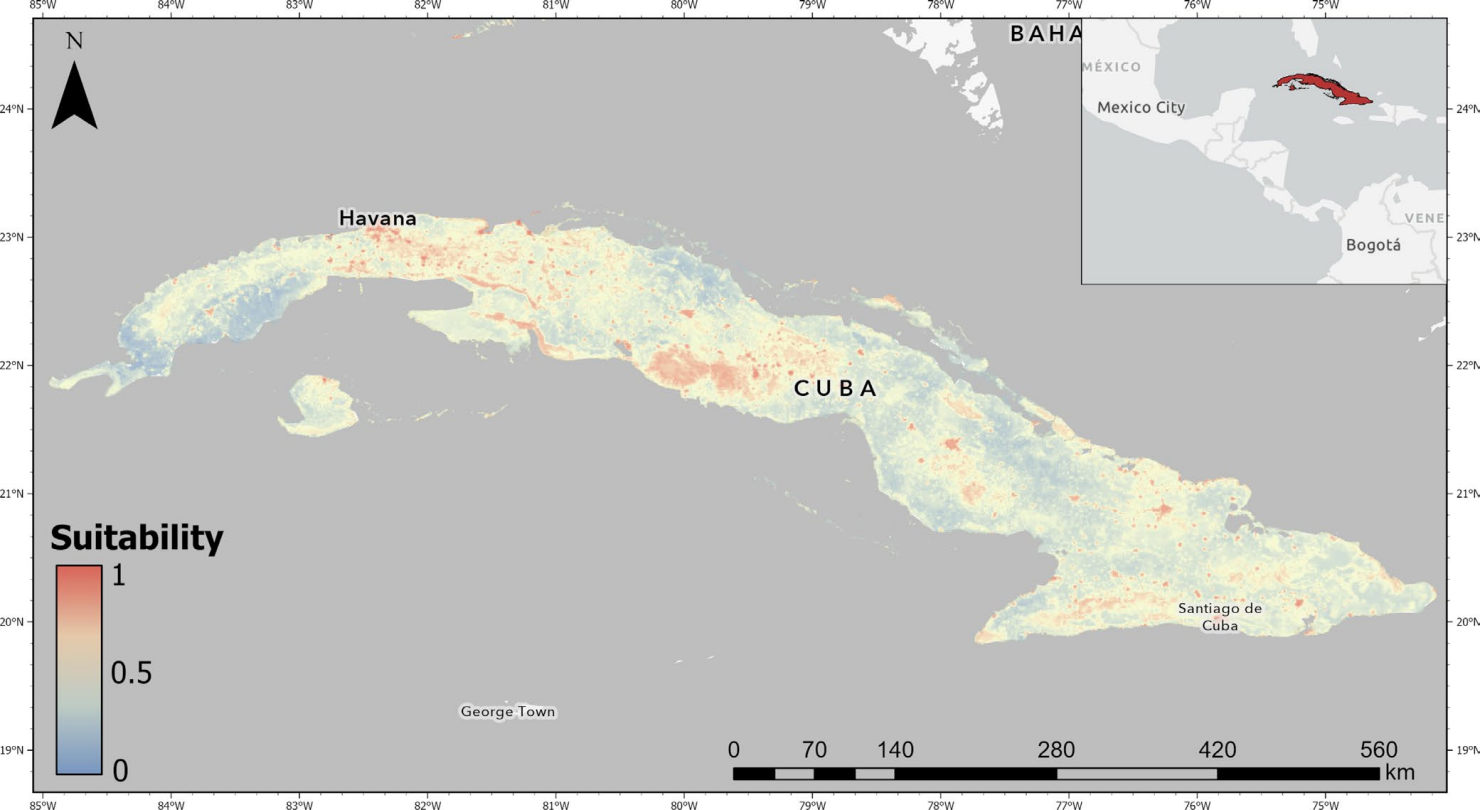
Habitat suitability for *Anolis equestris* across Cuba based on Random Forest species distribution model. Cooler colors (e.g., blue) indicate lower suitability, whereas warmer colors (e.g., red) indicate higher suitability. Raster resolution is 1 km^2^. Suitability values are scaled from 0 (unsuitable) to 1 (highly suitable).

The final model predicted suitability for *A. equestris* across much of western and central Cuba (Figure 2; Figure S4). Land cover in the western provinces, extending from La Habana through Matanzas, is dominated by agricultural practices and peri‐urban landscapes (Galford et al. 2023). Suitability was highest in the central provinces, Ciego de Ávila and Camagüey, where moist forests occur (Galford et al. 2023). In eastern Cuba, suitability included scattered patches in Santiago de Cuba and Holguín, corresponding to areas of moist forest (Galford et al. 2023). Low suitability was associated with regions dominated by mangroves, wetlands, pine forests, and dry scrub (Galford et al. 2023).

High suitability in Pinar del Río and Isla de la Juventud overlaps with the known range of *A. luteogularis* , a morphologically similar crown‐giant anole (Rodriguez Schettino 1999). Likewise, elevated suitability in parts of Holguín may reflect habitat occupied by *A. noblei* , another crown‐giant (Rodriguez Schettino 1999). Because our models approximate the realized environmental niche based on observed presences, such areas may reflect unmodeled biotic interactions.

### 3.3 Invasive Habitat Suitability

**FIGURE 3:**
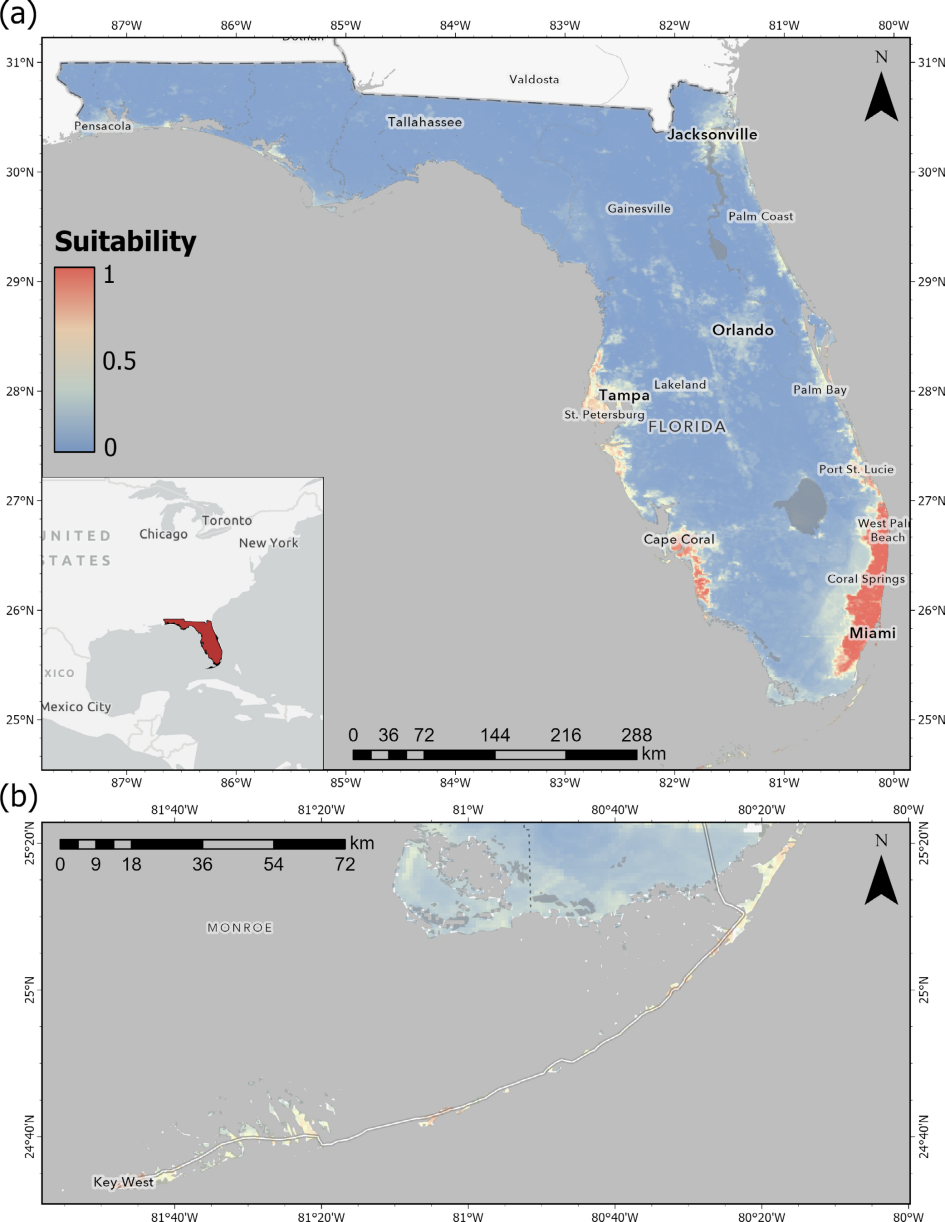
Habitat suitability for *Anolis equestris* across Florida. Cooler colors denote lower suitability whereas warmer colors denote higher suitability. Raster resolution is 1 km^2^. (a) An expanded view of peninsular Florida. (b) A detailed view of the Florida Keys.

The final model predicted a broad, nearly continuous band of high suitability for *A. equestris* along the Atlantic Coastal Ridge, extending from the Florida Keys through Miami‐Dade, Broward, and Palm Beach counties and tapering northward through Martin and into St. Lucie County (Figure 3a; Figure S4). Smaller clusters of suitable habitats occur on the southwest Gulf coast in Sarasota, Charlotte, and Lee counties. Suitability drops sharply from these areas of coastal development. Virtually all of Central Florida, Big Bend, and the Panhandle are classified as unsuitable, reflecting colder, drier conditions. However, some suitability is predicted within Duval County near Jacksonville. Currently, the northernmost record of *A. equestris* in Florida was recorded in St. Augustine in St. Johns County (Camposano and Krysko 2019), approximately 55 km to the southeast. Many of the Florida Keys, particularly those with greater human development (e.g., Key Largo, Marathon, Key West), had high suitability for *A. equestris* (Figure 3b).

### 3.4 Variable Importance

**FIGURE 4:**
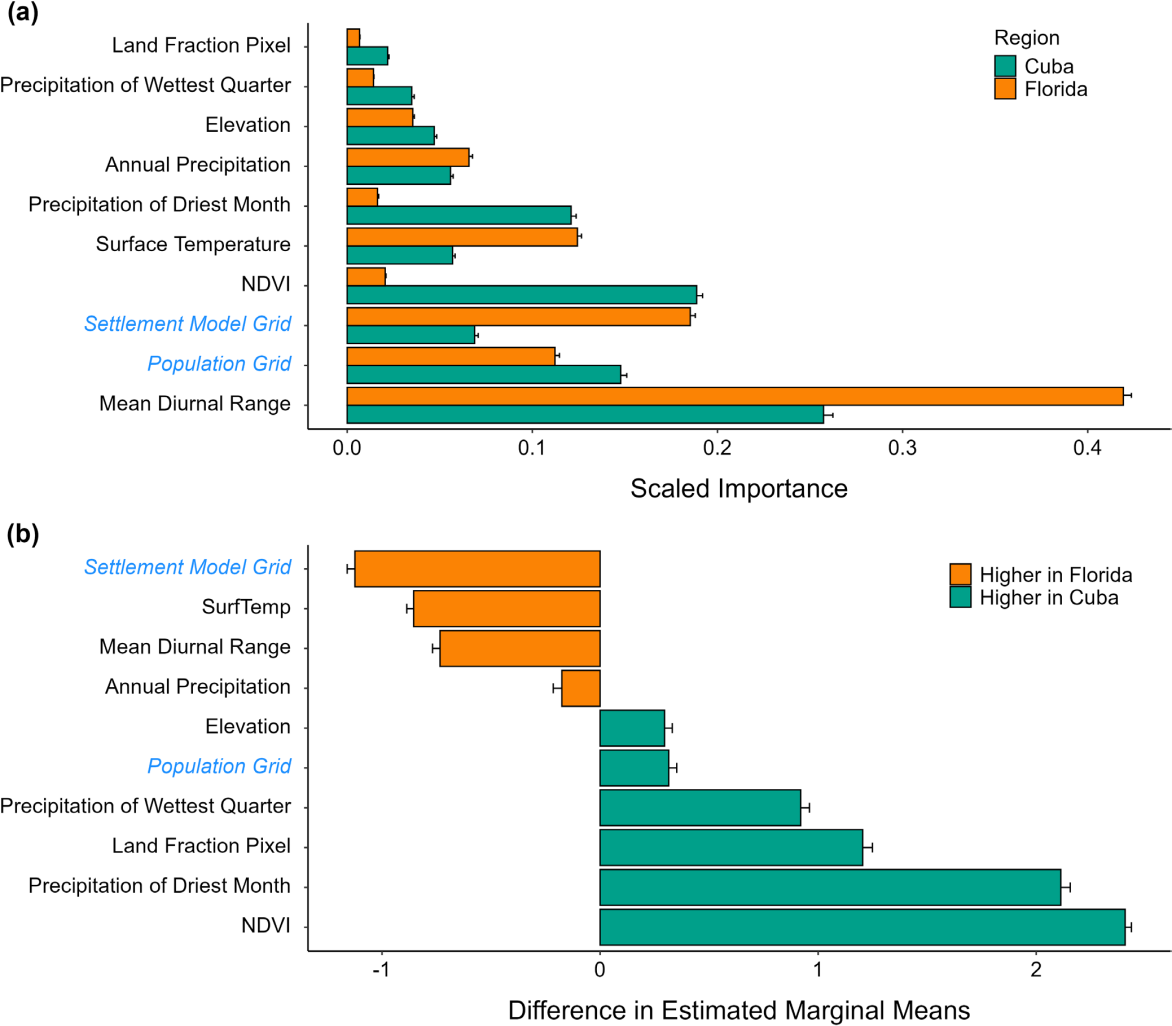
Variable importance of environmental predictors for species distribution model of *Anolis equestris* , by region. Variable names in blue denote anthropogenic predictors. (a) Mean scaled permutation‐importance scores for each predictor (± SE), averaged across all constituent model runs and validation replicates. (b) Pairwise differences (Florida–Cuba) of estimated marginal means (± SE) for each predictor on the logit‐link scale. Negative values indicate variables more influential in Florida. Positive values indicate variables more influential in Cuba.

Analysis of variable importance across the range of *A. equestris* (Figure 4a) identified mean diurnal range as the most influential predictor, with significantly higher importance in Florida than Cuba (contrast = −0.73, *z* = −41.78, *p* < 0.0001). Urbanization‐related variables, including the settlement model grid (contrast = −1.12, *z* = −62.19, *p* < 0.0001) and surface temperature (contrast = −0.85, *z* = −53.01, *p* < 0.0001), also had significantly greater importance in Florida (Figure 4b). In contrast, natural environmental features were more informative in Cuba. Notably, NDVI (contrast = 2.41, *z* = 164.76, *p* < 0.0001), precipitation of the driest month (contrast = 2.11, *z* = 95.63, *p* < 0.0001), and precipitation of the wettest quarter (contrast = 0.92, *z* = 44.67, *p* < 0.0001) were significantly more influential in the native range, reflecting the contribution of vegetation and moisture availability to native‐range suitability.

Additional regional differences were observed for elevation (contrast = 0.30, *z* = 16.36, *p* < 0.0001), land fraction pixel (contrast = 1.20, *z* = 52.92, *p* < 0.0001), and population density (contrast = 0.31, *z* = 16.77, *p* < 0.0001), all of which were more influential in Cuba. Annual precipitation had slightly greater influence in Florida (contrast = −0.18, *z* = −8.71, *p* < 0.0001). Collectively, these results indicate that habitat suitability in the native range is more strongly shaped by vegetation and precipitation regimes, whereas suitability in the invasive range is more closely associated with urbanization and temperature stability.

### 3.5 Threatened Invertebrates

**FIGURE 5:**
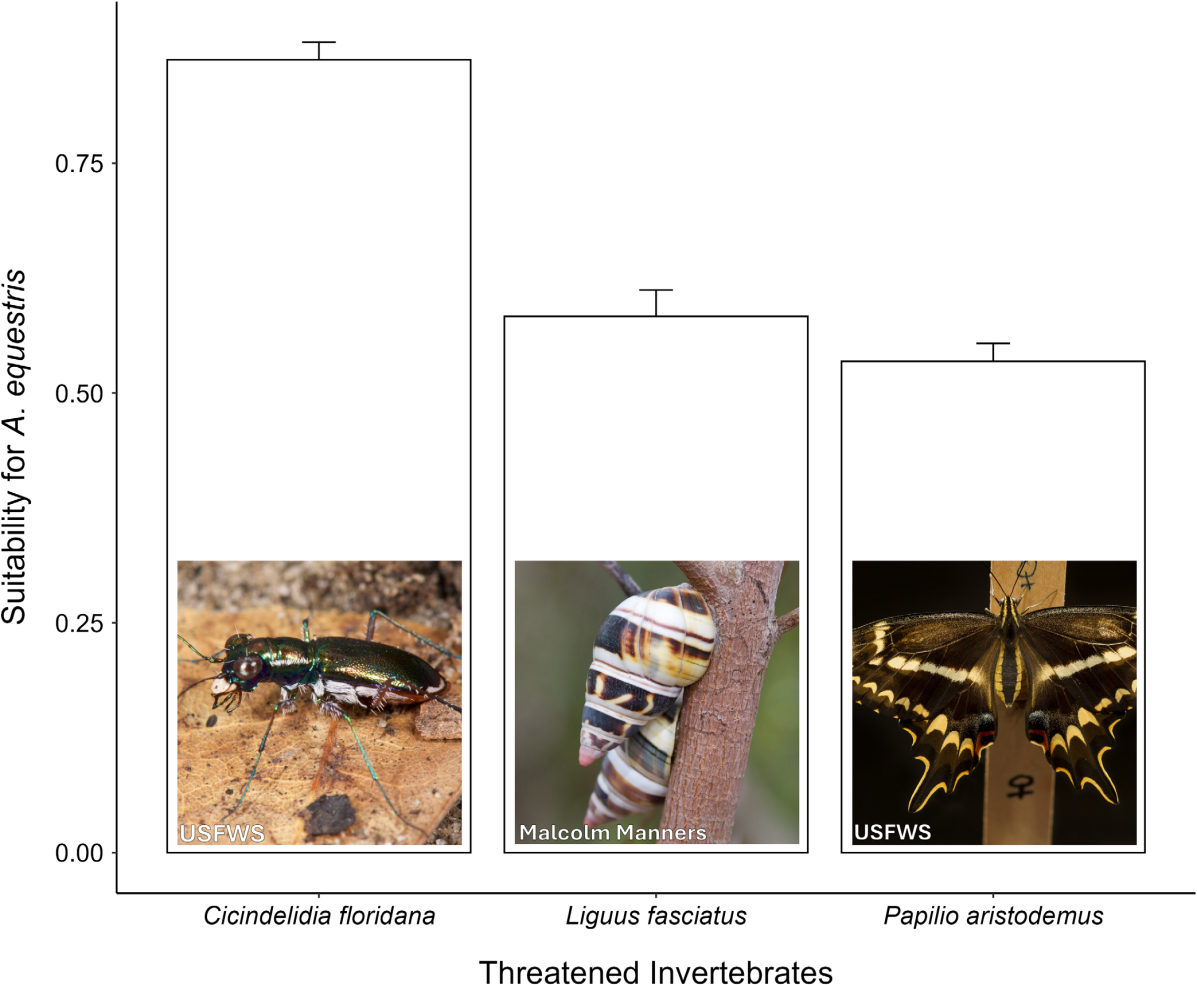
Habitat suitability of *Anolis equestris* at occurrence points of three threatened invertebrate (*Cicindelidia floridana*, *Liguus fasciatus* , *Papilio aristodemus* ) species in Florida. Bars represent *x̄* ± SE suitability values derived from Random Forest distribution model.

Mean habitat suitability for *A. equestris* in areas with the presence of *C. floridana* , an endangered tiger beetle, was 0.86 ± 0.02 (Figure 5). Mean suitability for *A. equestris* in areas with the presence of *L. fasciatus* , a tree snail and former species of special concern, was 0.58 ± 0.03. Mean suitability for *A. equestris* at the occurrence of *P. aristodemus*, an endangered butterfly, was 0.53 ± 0.02.

## 4 Discussion

In this study, we evaluated habitat suitability for *A. equestris* across its native and invasive ranges and predicted suitable habitats for *A. equestris* in both Cuba and Florida with high accuracy (TSS of 0.74 and 0.91, respectively). Additionally, we conducted a variable importance analysis, which demonstrated variability in the factors driving the distribution of this species between its native and invasive ranges. While climatic and vegetative factors were primary predictors of its native distribution, human development strongly influenced its invasive distribution. Specifically, coastal urbanized areas in South and Central Florida exhibited the highest habitat suitability. This has potential consequences for native biodiversity, including sensitive invertebrates such as *P. aristodemus* , *L. fasciatus* , and *C. floridana* , which may face predation pressure given the high predicted environmental suitability for *A. equestris* where these species occur.

*Anolis equestris* is strongly influenced by temperature and precipitation regimes, favoring warm, stable climates (Camposano et al. 2008). Temperature, particularly mean diurnal range, emerged as an important predictor of *A. equestris* habitat suitability. This effect was stronger in Florida, where increasing mean diurnal range at northern latitudes likely constrains the species distribution. Several bioclimatic measures of precipitation (precipitation of driest month, annual precipitation, precipitation of wettest quarter) were important predictors of habitat suitability across the range of the species, indicating their preference for mesic environments. Vegetative structure, as measured by NDVI, was moderately influential on model predictions, although it was significantly more influential on model predictions in Cuba. Thus, *A. equestris* is likely more reliant on natural habitats in its native range (Battles and Kolbe 2019). This is consistent with the finding that human development is a strong predictor of habitat suitability, and its importance is increased in Florida. Our results demonstrate that the ecological determinants of habitat suitability vary in the native and invasive ranges of *A. equestris* . Urbanization and thermal constraints dominate in Florida, while precipitation regime and vegetation‐related factors prevail in Cuba.

Our findings indicate that the Florida Keys exhibited substantial areas of predicted environmental suitability. Current field observations suggest that established populations are primarily concentrated on Key Largo, with fewer sightings on Big Pine Key and Key West. However, the model also identifies many intervening islands, particularly larger, heavily developed ones such as Plantation Key and Marathon, as environmentally suitable. While natural dispersal is likely constrained by water barriers, frequent vehicular movement and the ornamental plant trade could facilitate stepwise colonization (Krysko et al. 2011). Additionally, GBIF records as of December 04, 2024, indicate that *A. equestris* has been documented in nine additional counties beyond those reported by Camposano and Krysko (2019): Sarasota, Hendry, Manatee, Alachua, Charlotte, Highlands, Indian River, Lake, and Pinellas. Among these, Sarasota County had the highest number of observations (*n* = 14), followed by Hendry (*n* = 2) and Manatee (*n* = 2) counties. This expansion emphasizes the species' ongoing spread in Florida and highlights the need for sustained monitoring and targeted management efforts to mitigate potential ecological impacts.

*Anolis equestris* demonstrates a pronounced shift towards anthropogenic habitats in its invasive range (Camposano et al. 2008). However, rather than indicating a preference for urban environments per se, this shift may be better explained by the reduced competition and predation pressure in these areas. Urban environments likely emerge as key predictors not because buildings themselves provide essential habitat, but because they represent areas where these natural constraints are diminished (Eötvös et al. 2018). Additionally, artificial surfaces in urban settings may influence the species' thermal ecology, providing stable temperatures that support activity across a broader temporal window (Amadi et al. 2021). Nocturnal lighting could also extend foraging opportunities (Giery and Stroud 2013; Thawley and Kolbe 2020), while the presence of fruit‐bearing trees in human‐modified landscapes may enhance food availability (Giery et al. 2017). Given that *A. equestris* displays a broad tolerance of habitats in its native range (Rodriguez Schettino 1999), its transition into urban environments likely reflects this generalist strategy. These factors suggest that tolerance of human‐modified habitats in its native range may contribute to the invasiveness of *A. equestris* , warranting further investigation into how urban conditions facilitate their spread.

Native human association may represent a common exaptation found among invasive species. González‐Lagos et al. (2021) found that avian species which proliferate in human‐altered environments in their native range are more likely to be transported and introduced to new locations compared to species confined to wildland habitats. Furthermore, human‐associated birds had higher chances of becoming established due to their exaptations for coping with novel environments (González‐Lagos et al. 2021). Additionally, species that succeed in urban environments often exhibit specific adaptations, such as increased thermal tolerance and altered reproductive strategies, which enhance their invasion success (Borden and Flory 2021). In its native range, *A. equestris* occupies a wide variety of forested environments, with the exception of cloud and montane rainforests (Rodriguez Schettino 1999). The species is now common in fragmented urban–rural interfaces, such as suburban gardens, roadside vegetation, and cultivated landscapes, likely facilitating the transition to analogous habitats in Florida (de Andrade 2020). Given this, tolerance of human‐modified habitats by reptiles in their native range should be further investigated as an indicator of potential invasiveness.

The predicted habitat suitability of *A. equestris* in the range of threatened invertebrates raises concerns for their management. In Florida, *A. equestris* has been documented preying upon *L. fasciatus* (Giery et al. 2017). Florida tree snails were delisted in Florida in 2017 but are still considered imperiled and listed as an SGCN species. This species, along with the federally threatened Stock Island tree snail ( *Orthalicus reses* ), occurs in the southern portion of the *A. equestris'* introduced range. This overlap in range, as well as their arboricolous nature, is concerning as both of these tree snail species face major threats from another invasive species, the New Guinea flatworm ( *Platydemus manokwari* ). Other imperiled species of invertebrates, such as the federally endangered Bartram's scrub‐hairstreak butterfly ( *Strymon acis bartrami* ), *P. aristodemus* , and the Miami blue butterfly ( *Cyclargus thomasi bethunebakeri* ), are also facing threats like habitat loss and predation of host plants by green iguanas ( *Iguana iguana* ) (Krysko et al. 2007). Both *C. thomasi bethunebakeri* and *P. aristodemus* are currently undergoing reintroduction efforts in proximity to *A. equestris* populations (J. Parker, pers. comm.). Small species of birds, such as the state threatened white‐crowned pigeon ( *Patagioenas leucocephala* ), may also be prone to predation by *A. equestris* , especially during their hatchling phase. The overlap between *A. equestris* habitat suitability and the distribution of these threatened invertebrates suggests the potential for predation pressure facilitated by the high degree of urbanization in South Florida.

Given the documented predation on imperiled species and the overlap of *A. equestris* habitats with vulnerable native fauna, predictive tools like SDMs can facilitate the conservation of native biodiversity by mitigating the impacts of invasive species. Previous work has shown that SDMs can be used to identify the appropriate scope and scale of management efforts for invasive species (Lozano et al. 2024). Additionally, SDMs have been successfully implemented to guide monitoring efforts for invasive species (Meriggi et al. 2022). Thus, we believe our results can be used to guide targeted monitoring and removal efforts to minimize the impact of *A. equestris* populations on sensitive species. Conservation strategies should focus on limiting the overlap between *A. equestris* habitats and those of vulnerable species, minimizing predation risks.

We recognize that modeling with opportunistic occurrence data, even when rigorously filtered, carries unavoidable uncertainties. GBIF records vary in positional accuracy, observer effort, and taxonomic validation; although we screened coordinates, thinned presences to one per 1‐km cell, and incorporated an explicit effort layer, some residual bias almost certainly lingers. In Cuba, where museum specimens dominate and recent surveys are sparse, we retained several high‐uncertainty points to preserve geographic coverage, accepting a modest increase in error over the alternative of omitting large portions of the native range. The ten independent pseudo‐absence sets and 10‐fold cross‐validation scheme dampen stochastic artifacts, yet the background still depends on our stratified sampling design. Different choices might shift absolute metric values, even if the relative algorithm rankings remain stable. Importantly, external validation with a 15‐year monitoring program in Florida confirmed that predictive skill persists when transferred to data collected under systematic protocols, but similar benchmarking is not yet possible for Cuba. Finally, the ensemble projections approximate the realized environmental suitability of *A. equestris* under current climate, land cover, and propagule pressure. They do not account for future urban expansion, climate change, species interactions, or time‐lagged dispersal processes, any of which could alter the potential for *A. equestris* to establish in areas we presently classify as marginal.

Our focus on present‐day conditions reflects the study's aim to inform near‐term management for an established but still expanding invasion (Camposano et al. 2008; Camposano and Krysko 2019; Losos 2011). While future changes in urbanization, climate, species interactions, or dispersal could alter suitability patterns (Hui 2023), incorporating such projections would require scenario‐based modeling frameworks beyond our current scope. These extensions are important directions for follow‐up work once a robust present‐day baseline is established. Future research should also investigate the ecological interactions between *A. equestris* and native species, particularly along a gradient of anthropogenic disturbance, as such interactions may influence invertebrate community composition and associated ecosystem processes (e.g., pollination, nutrient cycling). Co‐occurrence modeling of threatened invertebrates and *A. equestris* could more precisely identify regions where predation pressure may be exerted on native biodiversity, thereby facilitating targeted management actions. Such modeling could also provide insights into habitat characteristics that influence these interactions, allowing for more nuanced management approaches.

Effective conservation requires targeted, evidence‐based interventions that directly address the results of this study. By focusing removal efforts and habitat management on regions of high habitat suitability and ecological impact, stakeholders can mitigate the ecological impacts of *A. equestris* . Our results highlight the importance of prioritizing management efforts for *A. equestris* at the urban‐wildland interface, where it is most likely to impact sensitive species. Additionally, we identified regions of South Florida, particularly the Florida Keys that possess high habitat suitability for *A. equestris* despite no current reports of its presence. This suggests that if the species reaches these areas, it is likely to establish unless management efforts are implemented to prevent this outcome. As community science observations were integral to this research, outreach efforts to increase public awareness and participation in reporting of the species can promote its proactive management. Furthermore, we determined this species displays tolerance of human‐modified habitats throughout its range, likely promoting invasiveness.

## Author Contributions

**Alexander S. Romer:** conceptualization (equal), data curation (equal), formal analysis (lead), methodology (equal), writing – original draft (lead), writing – review and editing (equal). **Sergio A. Balaguera‐Reina:** conceptualization (equal), formal analysis (equal), methodology (equal), supervision (equal), writing – review and editing (equal). **Eric Saurez:** conceptualization (equal), data curation (equal), methodology (equal), writing – review and editing (equal). **Edison D. Bonilla‐Liberato:** conceptualization (equal), formal analysis (equal), methodology (equal), writing – review and editing (equal). **W. James Whelpley:** conceptualization (equal), data curation (equal), methodology (equal), writing – review and editing (equal). **Frank J. Mazzotti:** conceptualization (equal), funding acquisition (equal), methodology (equal), supervision (equal), writing – review and editing (equal). **Melissa A. Miller:** conceptualization (equal), funding acquisition (equal), methodology (equal), supervision (equal), writing – review and editing (equal).

## Disclosure

Permission to reproduce material from other sources: All figures were generated by the authors or are reproduced under a Creative Commons license. The image of *L. fasciatus* is credited to Malcolm Manners, available at www.flickr.com/photos/mmmavocado/4216883441. The images of *C. floridana* and *P. aristodemus* are credited to the U.S. Fish and Wildlife Service Southeast Region, available at www.flickr.com/photos/usfwssoutheast/23408471139 and www.flickr.com/photos/usfwssoutheast/14359549885, respectively. All images are used under the terms of their respective Creative Commons licenses.

## Conflicts of Interest

The authors declare no conflicts of interest.

## Supporting information


**Appendix S1:** ece372334‐sup‐0001‐AppendixS1.docx.

**Appendix S2:** ece372334‐sup‐0002‐AppendixS2.xlsx.

## Data Availability

To protect the sensitive invertebrates analyzed in this manuscript, their occurrence data have been randomly displaced within a 10 km buffer of their original locations. Publicly accessible raster datasets (MODIS, WorldClim, GHSL, GMTED) and shapefile datasets (OCHA, FDOT) have not been distributed by the authors but are cited in‐text. All other data used in this analysis, along with the corresponding R script, are available in a public repository on Dryad (https://doi.org/10.5061/dryad.gqnk98szd).
