# Supplementary material for: Urbanization Drives Habitat Suitability of the Invasive Cuban Knight Anole (Anolis equestris) in Florida, USA
Source: Ecol Evol. 2025 Oct 14;15(10):e72334. doi: 10.1002/ece3.72334 (PMC12520795; doi:10.1002/ece3.72334)
Supplement: Supplementary file 1 — Appendix S1: ece372334‐sup‐0001‐AppendixS1.docx. [file ECE3-15-e72334-s002.docx]

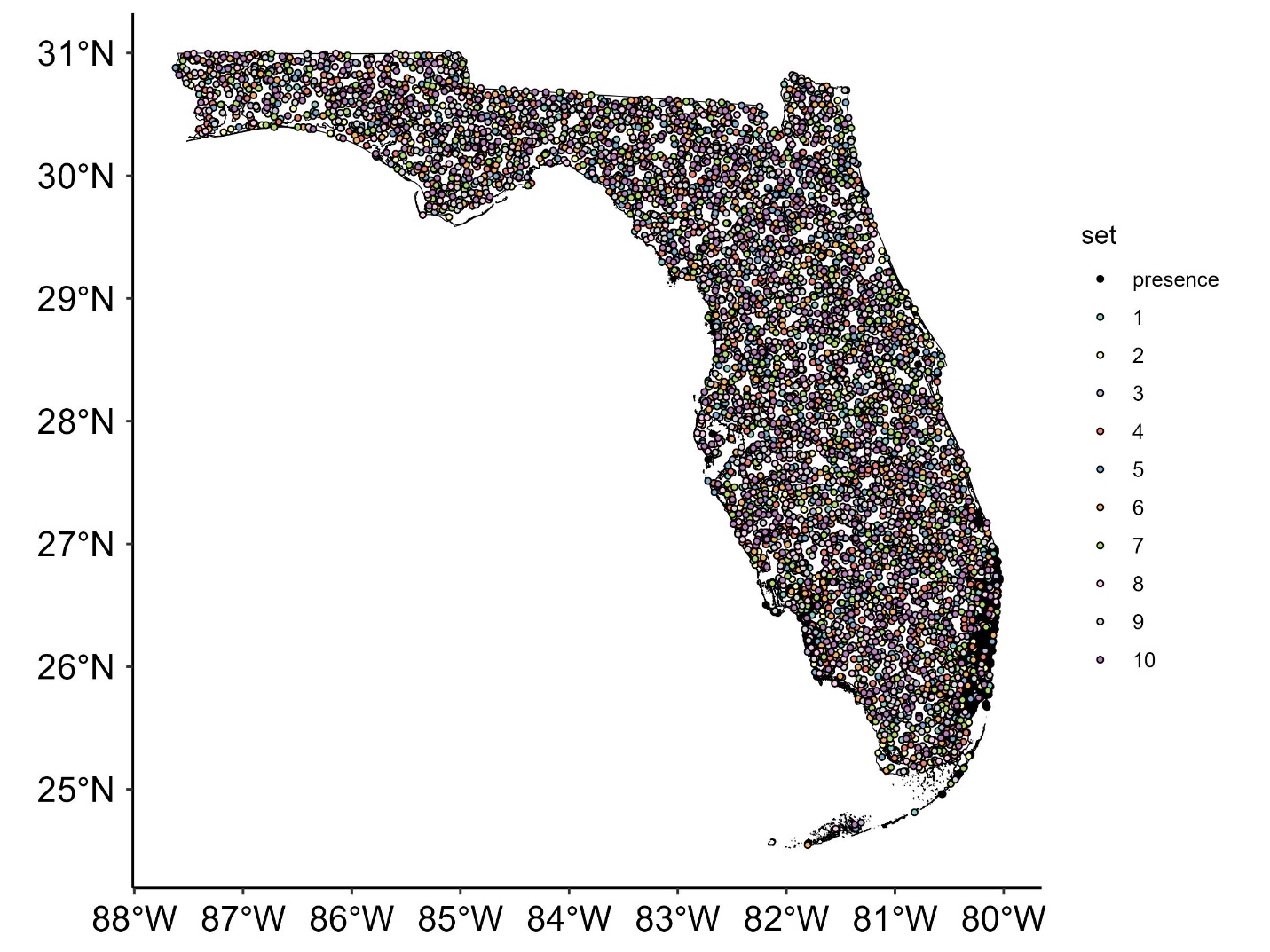


Figure S1. Spatial distribution of presence points (black) and ten pseudo-absence point sets (colored) used for species distribution modeling in Florida, USA. Each pseudo-absence set was generated independently to evaluate model robustness. State boundary is outlined in black.


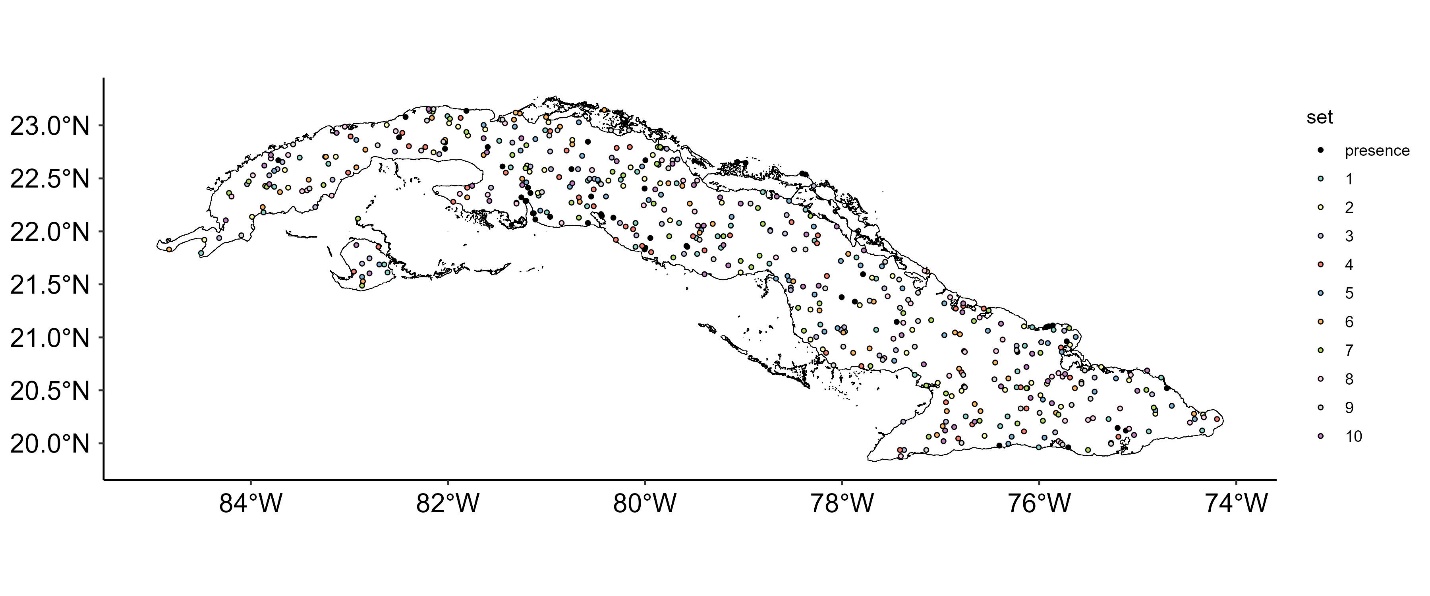


Figure S2. Spatial distribution of presence points (black) and ten pseudo-absence point sets (colored) used for species distribution modeling in Cuba. Each pseudo-absence set was generated independently to evaluate model robustness. National boundary is outlined in black.


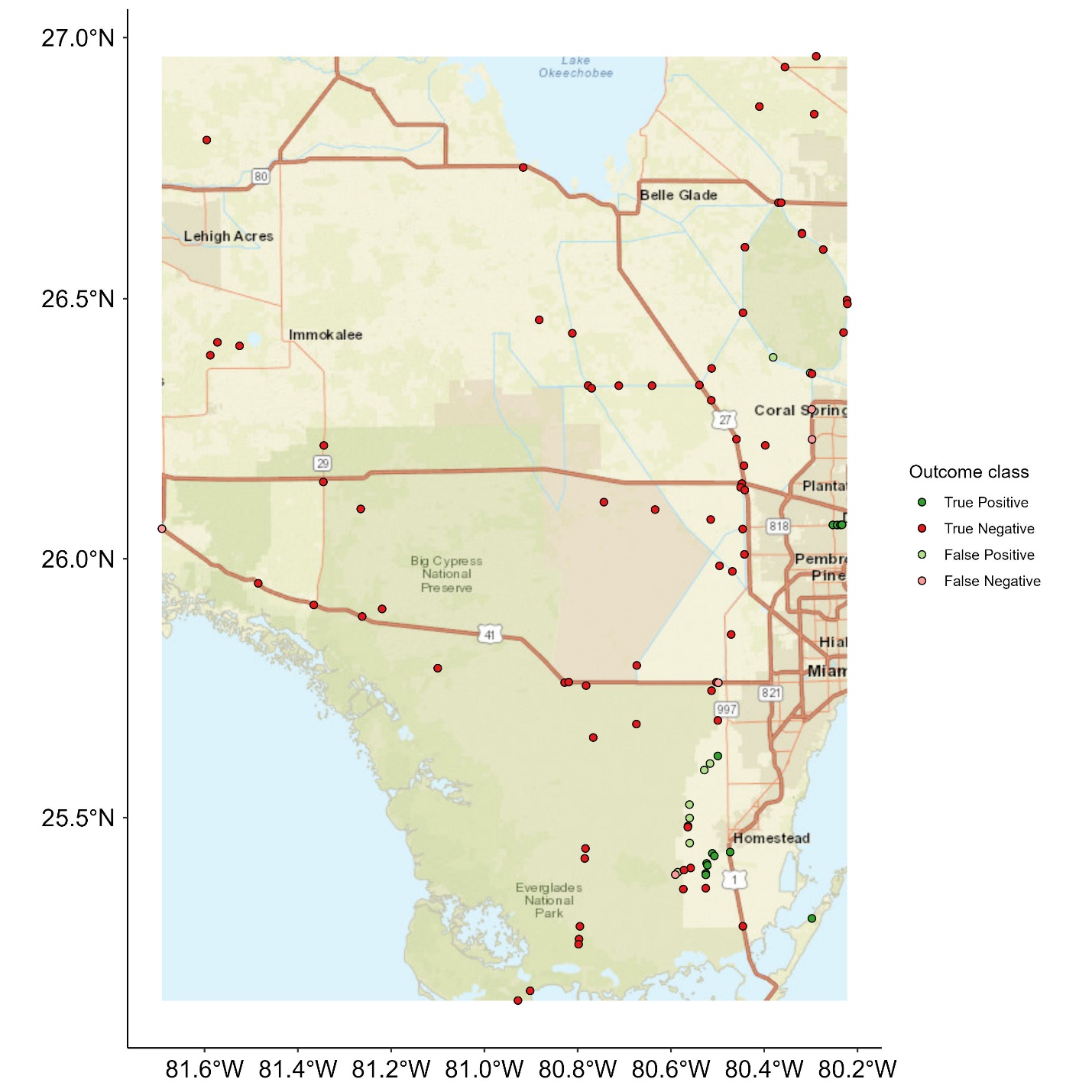


Figure S3. Independent evaluation of model predictions in southern Florida showing classification outcomes for test points: true positives (green), true negatives (red), false positives (pink), and false negatives (light green). The basemap is ESRI World Street Map.


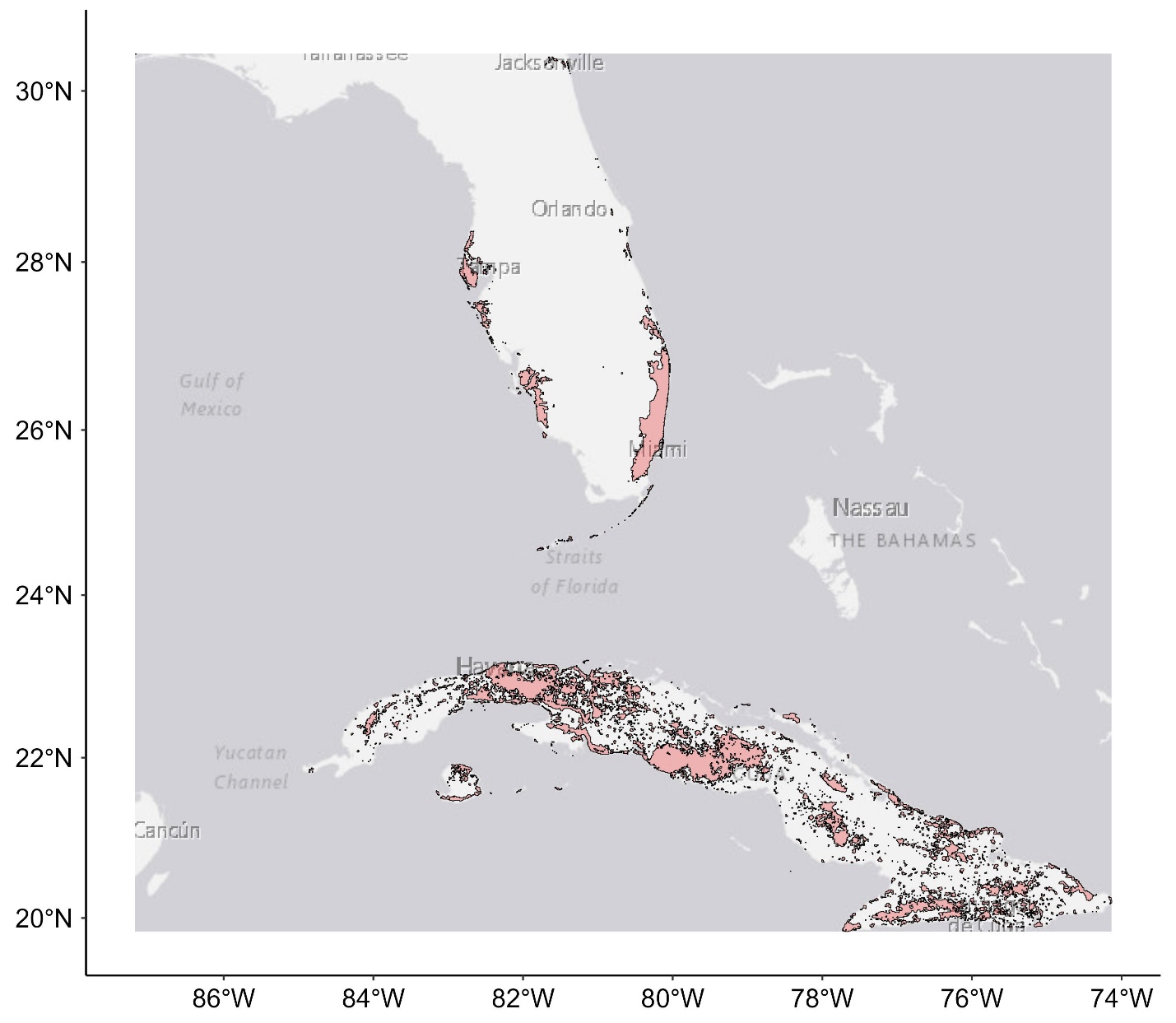


Figure S4. Binary habitat suitability (threshold = 0.5) projections for *Anolis equestris* across Florida and Cuba overlaid on the ESRI World Gray Canvas basemap. Pink shading indicates predicted environmental suitability
